# The meaning of postpartum sexual health for women living in Spain: a phenomenological inquiry

**DOI:** 10.1186/s12884-021-03578-y

**Published:** 2021-01-28

**Authors:** Lidia Pardell-Dominguez, Patrick A. Palmieri, Karen A. Dominguez-Cancino, Doriam E. Camacho-Rodriguez, Joan E. Edwards, Jean Watson, Juan M. Leyva-Moral

**Affiliations:** 1 Department d’Infermeria, Facultat de Medicina, Universitat Autònoma de Barcelona, Avda. Can Domènech, Edifici M. Despatx M3/213. Campus de la UAB, 08193 Bellaterra, (Cerdanyola del Vallès), Barcelona Spain; 2 Vicerrectorado de Investigación, Universidad Norbert Wiener, Av. Arequipa 444, 15046 Lima, Peru; 3 College of Graduate Health Studies, A. T. Still University, 800 West Jefferson Street, Kirksville, MO 63501 USA; 4 Center for Global Nursing, Texas Woman’s University, 6700 Fannin Street, Houston, TX 77030 USA; 5 Center for Qualitative Research, EBHC South America: A Joanna Briggs Institute Affiliated Group, Calle Cartavio 402, 15023 Lima, Peru; 6 Universidad Científica del Sur, Carr. Panamericana Sur 19, Villa EL Salvador, 15067 Lima, Peru; 7 Escuela de Salud Pública, Universidad de Chile, Independencia 939, Independencia, 8380453 Santiago de Chile, Chile; 8 School of Nursing, Universidad Cooperativa de Colombia, Calle 30, Santa Marta, Magdalena Colombia; 9 Nelda C. Stark College of Nursing, Texas Woman’s University, 6700 Fannin St, Houston, TX 77030 USA; 10 Watson Caring Science Institute, 4450 Arapahoe Avenue, Suite 100, Boulder, CO 80304 USA; 11 College of Nursing, Anschutz Medical Campus University of Colorado, 13120 East 19th Avenue, Aurora, CO 80045 USA

**Keywords:** Pregnancy, Postpartum, Sexual health, Sexuality, Body image, Social support

## Abstract

**Background:**

Sexual health is a multidimensional phenomenon constructed by personal, social, and cultural factors but continues to be studied with a biomedical approach. During the postpartum period, a woman transitions to mother, as well as partner-to-parent and couple-to-family. There are new realities in life in the postpartum period, including household changes and new responsibilities that can impact the quality of sexual health. This phenomenon is understudied especially in the context of Spain. The purpose of this study was to describe the lived experience of postpartum sexual health among primiparous women giving birth in Catalonia (Spain).

**Methods:**

This was a phenomenological study with a purposive sample of primiparous women. Data was collected through semi-structured interviews until saturation. Analysis followed Colaizzi’s seven-step process with an eighth translation step added to limit cross-cultural threats to validity. Also, the four dimensions of trustworthiness were established through strategies and techniques during data collection and analysis.

**Results:**

Ten women were interviewed from which five themes emerged, including: Not feeling ready, inhibiting factors, new reality at home, socio-cultural factors, and the clinician within the health system. Returning to sexual health led women to engage in experiential learning through trial and error. Most participants reported reduced libido, experienced altered body image, and recounted resumption of sexual activity before feeling ready. A common finding was fatigue and feeling overloaded by the demands of the newborn. Partner support was described as essential to returning to a meaningful relationship. Discussions about postpartum sexual health with clinicians were described as taboo, and largely absent from the care model.

**Conclusion:**

Evidence-based practices should incorporate the best evidence from research, consider the postpartum sexual health experiences and preferences of the woman, and use clinician expertise in discussions that include the topic of postpartum sexual health to make decisions. As such, human caring practices should be incorporated into clinical guidelines to recognize the preferences of women. Clinicians need to be authentically present, engage in active communication, and individualize their care. More qualitative studies are needed to understand postpartum sexual health in different contexts, cultures, and countries and to identify similarities and differences through meta-synthesis.

## Background

Sexuality is central to being human with an evolving meaning across the lifespan [1] that encompasses gender role, sexual identity and orientation, eroticism, pleasure, and intimacy [2]. Influenced by the dynamic interplay of variables [3] such as biological, psychological, social, economic, political, cultural, ethical, legal, historical, religious, and spiritual factors [4], sexuality is the integration of life experiences manifesting as a multidimensional phenomenon. Sexuality is embedded differently within cultures [5] through conventions, assigned roles, and behaviors that express sexual desires, diverse emotion and power relationships [6], mediated by the beliefs, values, attitudes, feelings and position in society [7]. As a subjective experience, sexuality depends on the person, and usually their partner, in the context of bodies, feelings, and emotions [8]. Sexuality emerges as what has been learned culturally, historically, and socially without an emphasis on the biomedical roles of biology and reproduction [9]. In the context of women, however, sexuality is influenced by significant life events and experiences associated with pregnancy, childbirth, and motherhood [10, 11].

### Sexual health and women

Recognized as a basic human need to honor and celebrate the essence of humanity, sexuality is a central concept for sexual health [12], with least three related functions: reproduction, pleasure, and communication [13]. For a woman, sexual health is expressed in the sexuality constructed by personal, social, and cultural factors. Since the early 1970’s, sexual health began shifting from a biomedical phenomenon defined by physical properties to “the integration of the somatic, emotional, intellectual and social aspects of sexual being in ways that are positively enriching and that enhance personality, communication and love” [14]. Today, sexual health is “a state of physical, emotional, mental and social wellbeing in relation to sexuality; it is not merely the absence of disease, dysfunction or infirmity” [15]. With this evolution, sexual health promotion was one of the five core reproductive and sexual health services included in the Millennium Development Goals [16], and carried forward into the Sustainable Development Goals [17]. As a basic human right [18], women have the right to pursue a fulfilling, satisfying, safe, and pleasurable sexual life [19, 20].

The trajectory for sexual health during motherhood begins with pregnancy, progresses to birth, and evolves through postpartum. Despite the postpartum period being largely defined by physical changes across the three phases emphasized in the biomedical model of childbirth, the new mother also experiences psychological, relational, and social changes [21, 22]. Although the postpartum period is characterized by similar physical changes, the return to pre-pregnancy sexual health is individual, personal, and changes with time [23]. Complications, such as pelvic floor disorders [24–26], can negatively affect the sexual health of primiparous women with recovery taking between 3 and 18 months [27–30]. The longer recovery often results in decreased emotional satisfaction, new relationship problems, and inadequate sexual pleasure [31].

### Postpartum sexual health

Conceptually [8] and clinically [11], postpartum sexual health is not well defined and lacks sufficient published research to be well understood. As a concept, postpartum sexual health has been described by four interrelated dimensions, including: physical, psychological, relational, and social [8]. For women in varied contexts, unique cultures and different countries, shifting roles and evolving responsibilities in the postpartum period negatively impact their sexual desire as well as satisfaction [32–44], and even stimulates fear [45]. Researchers, primarily from economically developed countries, have reported a variety of issues that impact the overall experience of women when returning to sexual activity, including: dyspareunia [22, 34, 36, 40, 41, 46–52], decreased libido [51, 53–55], problems achieving orgasm [11, 46, 48, 56], negative body image [32, 53, 54], fatigue and sleep deprivation [53–55, 57], relationship dissatisfaction [43, 57], household responsibilities [54], and concern about satisfying partner [22, 39, 42, 43, 46, 48, 58, 59]. Also, women report emotional concerns and personal conflicts [50, 53, 54, 60, 61], such as feeling guilty for their problems and failing their partners [51, 53, 54] and even depression [34, 46, 55, 57, 62]. These sexual health issues are reported to persist for 12 months and longer following birth [11, 38, 40, 41, 56].

Sexual health has been described as a taboo topic for conversation for women seeking health services [58, 63]. While some women reluctantly engaged their healthcare providers in discussions about sexual health [39, 47], other women, as reported in Spain [33], lacked the prerequisite knowledge about sexual health to seek help during the postpartum period. When seeking help from their providers, women believed that their concerns were trivial, or the information they received was not helpful [64]. More adaptative to changes in their sexuality than their partners [65], women in the postpartum period hesitantly return to sexual activity without guidance from their providers to satisfy their partners or to feel attractive and desired [43, 59]. Regardless of the explanation, the available evidence suggests increased couple time, effective communication, partners engaged in newborn care, and shared household responsibilities [53, 54, 60, 61] decreases conflicts and increases intimacy resulting in improved postpartum sexual experiences.

More qualitative research is needed to address the ‘dearth of evidence’ [11] about how women experience their sexual life during the postpartum period. This is especially important since researchers report sexual activity is not delayed due to delivery type, whether vaginal with or without episiotomy or caesarean, and laceration status [38, 39], even at 12 to 18 months postpartum [37, 41]. Sexual health cannot be studied in isolation as a physical concept [59]; instead, it should be explored as a multidimensional phenomenon focused on the ‘whole woman.’ As such, clinicians need to assess, identify, and intervene to improve the sexual health of individual women, taking into consideration their perspective, as part of holistic care [66].

In the Spanish context, there are five studies reported in the literature that explore any aspect of postpartum sexuality [32–35, 67], with minimal focus on sexual health. Two of the Spanish postpartum and sexuality studies used observational methods to understand the mode of birth and postpartum sexual functioning in Madrid [34] and self-esteem and self-image of women in Navarra [32]. The other three studies were ethnographies focused on sexuality and religiosity/spirituality in Castilla-La Mancha [35], sexual experiences of Spanish and immigrant women in Palma de Mallorca [67], and the expression of sexuality socially and professionally in Madrid [33]. There were no studies specific to sexuality or sexual health identified for the distinct culture in the large region of Catalonia (Spain). This phenomenological research was conducted to explore and describe the lived experiences of postpartum sexual health of primiparous women in Catalonia (Spain).

## Methods

### Design

A phenomenological method [68] was used to answer the research question for the study: What is the meaning of sexual health for women during the postpartum period? Descriptive phenomenology guides researchers to capture the reality of phenomena through probing the subjective consciousness of the individual [69]. This approach is commonly used to study poorly understood aspects of experiences [70]. For this reason, this study used participant subjectivity to illuminate the meaning of their experiences as they were experienced [71]. The study was approved by the Research Ethics Committee of the Universitat Autònoma de Barcelona (Protocol #3647).

### Sampling and participants

A purposive criterion sampling technique [72] was used to identify women who experienced the phenomenon of interest [73]. The recruitment was assisted by a midwife practicing in Tarragona (Southern Catalonia) who informed potential participants about the study. Contact details were collected from the women who expressed interest in the study. To be eligible to participate, women had to have given birth for the first time (primiparous) within the last year in a Catalonian hospital. Women were excluded if they were < 18 years old, gave birth to more than one infant (twins or triplets), did not speak Catalan or Spanish languages, or had cognitive disabilities. The women were contacted by telephone by the principal investigator and if they agreed to participate, a specific date and place was arranged for the interview. Skype® was also offered to participants as an interview format to facilitate recruitment [74]. The participant enrolment process was closed when the interviews provided no new information, or the information was redundant to the point of data saturation [75, 76]. Saturation was anticipated to occur between six and fifteen participants [71, 77–79].

### Data collection

Data were collected in Catalan or Spanish through semi-structured interviews with the participants between March and June 2017. The primary investigator conducted the digitally recorded interviews as recommended by Rubin & Rubin [80] to help the women express themselves freely in describing their experiences and identifying their perceptions about their postpartum sexual health. The interview guide developed for this study focused on exploring six areas referenced in the literature [1, 8, 10], including: 1) resuming sexual activity; 2) experiences; 3) emotions and feelings; 4) role of the partner; 5) influence of the infant; and 6) support from clinicians (health professionals). The interview questions (Table 1) facilitated a conversation where the researcher listened to the participants describe their lived experiences [80]. In order to explore the six areas of interest, the researcher minimally guided [81] rather than directed or controlled [82] the conversation. As new areas emerged, such as the significance assigned to sexuality by society, the topics were incorporated into the continuing interview process [83]. Each participant signed an informed consent, approved by the research ethics committee, prior to beginning the interview. Participants were able to withdraw at any time during the study. Anonymity was assured by replacing real names with pseudonyms and no financial incentives were provided to the participants.

**Table 1:** Questions for semi-structured interviews

• In general, how would you describe your sexuality? What is the meaning of sex for you?	
• What was your sexual experience the year prior to pregnancy?	
• In what ways did you think having a child would impact your sexuality and your sex life?	
• At what point did you feel ready to return to sex? Why? What prompted this decision?	
• How would you describe your ability to manage things after the baby was born? What support did you received from your partner?	
• What has been the impact of childbirth on your relationship? How does sex impact your partner?	
• What physical changes to your body did you notice most during pregnancy? How did these changes impact your sexuality? Do you have any concerns now? Tell me more … ..	
• What is your motivation for having sex now? Has this changed from the past? Tell me more … ..	
• When thinking about the care provided by health care providers during your pregnancy, what was their role in taking care of your sexuality? Before birth? After birth? Now?	
• Is anything else that you would like to discuss?	

### Data analysis

The data were manually analyzed in Spanish with the seven-step method described by Colaizzi [84], including: 1) familiarization, 2) identifying significant statements, 3) formulating meanings, 4) clustering themes, 5) developing an exhaustive description, 6) producing the fundamental structure, and 7) seeking verification of the fundamental structure. As the data were analyzed in Spanish but needed to be reported in English, an eighth translation and interpretation step was added to the method. This included a qualified native bilingual translator (Spanish-English) producing an English language final report with verbatim data [85, 86] and an external bilingual expert review of the final codes, categories, and exemplar quotes [87]. Ten women were interviewed before reaching data saturation. Each interview lasted about 45 min (ranging from 31 to 56 min), with eight conducted in person and two conducted via Skype®.

### Trustworthiness

To ensure the trustworthiness of the data, methodological strategies to establish credibility, transferability, dependability, and confirmability guided the process [88, 89]. The strategies incorporated into this study included audit trail, bracketing, coding checking, categorizing, constant feedback, continual data interaction, participant confirmation, peer debriefing, structural corroboration, and referential adequacy [90–93]. The data translation was completed in an iterative process including continual discussion with the research team to preserve data integrity [85]. In order to maintain the authenticity of the meaning for dissemination [86], the source and target language codes, categories, and exemplar quotes were reviewed by three external bilingual experts. Finally, the 32-item Consolidated Criteria for Reporting Qualitative Research [94] checklist was applied to this study to provide a thorough, transparent, and trustworthy account of the data collection process, analysis, and the relationship to the findings.

## Results

All ten participants were primiparous heterosexual women, mostly with professional employment and living in Tarragona (Catalonia, Spain), with an average age of 33 years (range of 28 to 36). The complete demographic data for each participant is provided in Table 2. From the transcribed data, a total of 207 descriptive codes were identified. After grouping these codes by similarity, interrelated themes emerged from the data. The five themes that described the meaning of postpartum sexual health, included: 1) not feeling ready, 2) inhibiting factors, 3) new reality at home, 4) socio-cultural norms, and 5) clinicians within the health system. To evidence these themes, exemplar quotations from the participant interviews were selected and provided by theme in the analysis below.

**Table 2:** Participant Characteristics

Participant	Age	Civil status	Infant age	Intercourse after birth	Episiotomy	Baby feeding
Georgina	35–39	Cohabiting	3 months	40 days	yes	Breastfeeding
Irene	30–34	Cohabiting	6 months	60 days	yes	Mixed lactation
Raquel	35–39	Married	7½ months	105 days	yes	Artificial lactation
Laura	30–34	Married	7½ months	60 days	yes	Artificial lactation
Silvia	30–34	Cohabiting	5 months	Not yet	yes	Breastfeeding
Patricia	25–29	Cohabiting	6½ months	60 days	yes	Artificial lactation
Olivia	30–34	Cohabiting	11½ months	30 days	no, Caesarean	Artificial lactation
Vanesa	30–34	Cohabiting	4 months	45 days	yes	Artificial lactation
Esther	30–34	Married	4 months	90 days	yes	Breastfeeding
Ruth	35–39	Married	4 months	Not yet	yes	Breastfeeding

### Not feeling ready

The theme ‘not feeling ready’ refers to the wide array of emotions and feelings women experienced after giving birth, either in relation to their sexual health, motherhood, or the new relational context. Almost all the women reported being willing to and trying to have intercourse with reservation about their readiness. They described being motivated by the desire to return to what they perceived to be the ‘normal’ (pre-pregnancy) way in relationships. Although they did not feel directly pressured, some women stated they wanted to please their partners, so they did not always feel rejected.

"You have to have willpower, we have to try to do it at least once a week because otherwise it [intercourse] will be more difficult, right? […] Because later it will be worse. Yes, you must make an effort" (Irene).

Almost all the women reported they had no sexual desire; describing their return to an active sexual relationship as complicated and scary, especially because they feared the unknown including the prospect of physical pain with intercourse. Several women reported they dwelled more on their fears rather than the possibility of enjoying sexual activity. Half the women confessed they did not feel ready to resume intercourse, either due to physical and/or emotional reasons.

"Okay, I still do not feel one hundred percent ready, and except for the last two times, which have been normal, with the rest I had to be careful because I still had difficulties” (Raquel).

The loss of libido, changing roles, breastfeeding, awareness of the newborn, and feeling that the ‘entire weight’ of the new family fell on them, contributing to the women feeling sad, seeking to escape, and thinking they were unfit mothers. The women felt alone as their husbands returned to work, increasing their sense of overload and their insecurity about not knowing how to care for their newborn.

"At first, I told him, I was very happy before [having the baby]. I could not deal with everything. The first month and a half I was sunk; the whole day having the baby hooked to my breast, I was alone, he went to work right away, you know?" (Vanesa)

To mitigate these feelings, the women needed to find a moment for themselves to feel liberated; to recover their physical and mental energies. Almost all the women reported they were able to cope with their situation when their partners were understanding and supportive. Feeling respected and encouraged by their partners gave the women a sense of security, a peace of mind.

"My husband supports me in all of this. He is a very quiet person, you know? He helps me with the baby […] He understands that there are times when I do not feel like it [having sex] or that I am not motivated. And yes, he gives me all the support" (Laura).

### Inhibiting factors

The ‘inhibiting factors’ describes the physical experiences of the women during the postpartum period, especially during ‘*la cuarentena*”, a culturally-based family ritual to support the new mother during the 40 days following birth that includes no sexual activity [95, 96]. In terms of resumed sexual activity at the time of their interview, only two women remained celibate. Nevertheless, their experience was congruent with the other women. In general, the women described the pain and discomfort they experienced with the first vaginal penetration. Almost all participants highlighted the principal problem as the episiotomy stitches hampering their return to sexual activity.

"Resuming sexual intercourse was hard, very hard. The truth is that ... it was very difficult for us. Firstly because, well, I do not know if it was normal or it was not normal, but the stitches took a long time to reabsorb and everything really hurt” (Raquel).

Other problems described by the women were bleeding, feeling dirty, pelvic floor problems, and sensitivity alterations.

"I was not in the mood for sex. It was because of the bleeding... I felt dirty, it was very unpleasant. You have the period every month and it is uncomfortable, but this [postpartum] it was diferent, harder. I was thinking all the time that I needed to wash myself down there” (Patricia).

The women associated sexual activity as intercourse; indicating there was no foreplay or other physical contact with their partners or with themselves. Most of the women had no sexual desire; reporting vaginal dryness and other physical changes that hampered intercourse. The two women who reported feeling sexual desire following childbirth, also were less concerned about their physical changes. The women indicated a lack of time and decreased libido led to “fast sex”, with no prior stimulation. The women recognized this might have limited their sexual desire.

"During the ‘cuarentena’? No, not by any means, not desire, not motivation. Following the ‘cuarentena’, it took us a while to start having sex. I’ve noticed that I do not have as much desire as I used to; before giving birth, we had sex at least once a week, and now maybe once every other week or every 3 weeks. I think it’s because of the libido, I don’t know where it’s gone [laughs]” (Laura).

The women explained they had to go slowly with intercourse, trying to move into ‘gentle’ positions, and stopping when they felt unable to continue. Both the rhythm of the sexual relationship and the frequency of intercourse changed; however, the women felt this was common while worrying they would not recover to their previously satisfying sexual health.

"We went very little by little, without doing any uncomfortable positions. I didn’t feel bad at any time; I knew if it went wrong, I could stop and try again. Finally, it was good, very good indeed” (Patricia)

The physical changes associated with childbirth led to negative reports of sexual health during the postpartum period. Although the women did not report feeling rejected by their partners, they were acutely aware the physical changes (e.g. round belly and stretch marks) negatively impacted their self-image, made them feel less attractive, and reduced their libido. The women also stressed they perceived their breasts much differently; previously a symbol of femininity and sensuality, they were basically a ‘feeding machine’ for the baby with almost no sensitivity.

"Besides all your body changes, there are also the breasts; they are not as they were before giving birth. You are there the whole day watching your baby suckling and … that negatively influences your libido, for sure. Now when my partner touches them, he does it in another way it's … it's weird, now it's weird" (Esther).

### New reality at home

The ‘new reality at home’ theme describes the impact of the arrival of the newborn regarding changes in family roles, distribution of household responsibilities, care of the newborn, and the sexual relationship. More than half the women reported their relational priorities significantly shifted after childbirth. The women devoted most of their time to caring for their baby; contributing to the feeling of losing their womanhood and producing the nostalgia for needing their personal space. Following the birth, the women felt overloaded until they found a harmony with their baby. Many women used the expression “*24/7*”, referring to the total dependence of their baby, which caused distress. They highlighted the need for balance in caring for their baby and attending to their partner; achieving this relational balance required mutual support and reciprocal collaboration.

"We want the baby a lot and, but you know? You have a child, fact; but you also have a partner, right? We are aware that the baby will always be here, and we will give him what is right and everything, but we also need to have this “couple moment” (Patricia).

In addition to reducing sexual desire, breastfeeding was also described as a stressful and worrisome burden. The women wondered if they were correctly feeding their babies. And, they felt completely ‘tied down’ by breastfeeding, their exclusive responsibility. In contrast, the women fondly described the intensely close bond that emerged between themselves and their baby while wondering with frustration where is their partner in this picture.

"And he [partner] seemed to have no child, and even worse, as breastfeeding is ‘a maternal thing’, he was there as a movie extra. When breastfeeding came to an end, oh gosh! What a change! What a relief!” (Raquel).

The inability to sleep at night was highlighted as a critical problem by all the women. Those who could rest properly felt more energetic, both regarding their ability to engage in sexual activity as well as care for their baby. Rest required support from their partners, while good communication was important when the women felt tired. However, the communication was not always good as the loss of their role as a woman coupled with being a new mother led some couples to distance themselves from each other. There were more arguments, especially about the distribution of household tasks and the care of their baby. Some women reported feeling that they were the only one leading the way while their partners were merely bystanders. The lack of personal time and the distancing required couples to ‘book’ a time for intimacy. This translated into scheduled versus spontaneous sexual activity, further hindering their sexual desire. Finally, more than half the women felt the presence of their baby in their bedroom was a negative factor for intercourse. With the baby present, the women felt uncomfortable as the total loss of privacy resulted in feeling less intimate.

"The crib is in the next room, there's no privacy, you know? We're tired, we want to go fast and of course, it [having sex] takes time" (Ruth)

### Socio-cultural norms

There were beliefs and preconceived ideas the women mentioned as being part of their social and cultural backgrounds which influenced their sexuality after childbirth. All the women stressed they avoided preconceived ideas about sexual health during the postpartum period to experience it calmly and naturally.

"And the best thing you can do for everything, is to carry nothing, no preconceived ideas. But nothing uh, neither during pregnancy, nor in childbirth, nor in the postpartum, nor in relationships, nothing. That is, whatever happens you will manage it the best you can.” (Raquel).

The women felt the pressure exerted on them by society was unhelpful in dictating how they should live and in what way they should behave as a mother. They felt all the responsibility for the household was theirs. The society expected them to know how to do everything and how to satisfy everybody except themselves. This pressure provoked tiredness and negatively affected their sexual health.

"And on top of that [becoming a mother] you have to know everything, that is, you have to be a mother, a woman, a housewife and a friend, and you have to be perfect and stupendous at all times, and this is hard. Am I supposed to be ready for … [sex], you know? When I am exhausted? Excuse me, but no! The mother is in charge of everything, no matter if you are young or old, if you are expert or novice … it seems that you have to know from the first day how to change a diaper!" (Raquel).

Some of the women mentioned the difficulties and negative parts of motherhood, especially in relation to their sexual health, were not explained by anyone. They reported everyone tended to idealize the arrival of the baby or overlooked the difficult situations. When the preconceived ideas and incorrect expectations clashed with the reality of their lived experience, the women felt as if they were doing things wrong. All the women agreed the discussion of postpartum sexual health was taboo, as the topic provoked shame in both the mothers and the other people in their world. The women argued more information needed to be shared with them, including life experiences from other mothers, to understand the meaning of being the new mother in the context of being part of a couple.

"It is not difficult for me to talk about sex, but I do believe that there are people who are quite shy in this regard, not me I repeat. Your friends and relatives don’t explain anything to you about sex, they may want to, but they don’t do it” (Esther)

On the other hand, having the support of friends and other mothers helped the women to have a better experience, and to understand what was happening by giving them an opportunity to exchange information freely among equals. Sharing their own experiences reassured them of their normalcy. Interestingly, more than half the women tended to seek normalcy, defined, in their own words, as “*what others do*”.

"If you go and someone tells you ‘it was the same for me too!’ or ‘I don’t know if that is normal either’, it's like you go home more calmly, because I'm not the only one, right? (Esther).

Another relational factor identified in the collective experiences of the women was their personal interpretation of sexual health; this was quite similar for all of them. The women emphasized their need to connect with their partners positively and actively. Although they wanted to have sex with their partners as in the past, this was difficult after childbirth since the women needed to prioritize their baby.

"It [having sex] was one of the hardest parts of the difficulties of having a child, I’d say the hardest. You think that everything is going to be perfect after having the baby, but it turns out that it's not all that perfect and sex, at all!" (Raquel).

### Clinicians within the health system

Finally, the women felt their experiences with the health system in the postpartum period could have been much better. Most participants confessed to having personal doubts in the postpartum period, mainly about their ability to care for their baby. Although all the women attended postpartum maternal education classes, they felt lost and alone; not only in relation to caring for their baby, but also in resuming sexual activity.

"Yes, at some point I had many doubts, too many. I became an expert in reading, oh my God! Anything that happened to the baby, anything that I experienced looking after him … it was huge. You have many, many doubts. You are completely lost" (Ruth).

The women recognized their exclusive interest in caring for their baby resulted in neglecting themselves and experiencing postpartum sexual health in an unexpected way. They believe the clinicians should be responsible for preparing them to manage the changes they would experience. The women wanted to talk openly, explicitly, and without generalizations, with someone about their sexual health in the postpartum period. Above all, the women wanted their clinicians to be honest and direct about their sexual health, without glossing over the negative aspects and addressing the real difficulties they would most likely experience following childbirth.

"They [clinicians] prepare you for the postpartum, yeah, but just physical things down there, nothing about sexuality. They told us before we left hospital, that you may have a bad time, you may cry, but I didn’t think it would be like this" (Vanesa).

Several participants stated that they preferred to consult the internet instead of their clinicians as their primary source of information.

The women emphasized that their need to share their sexual experiences with their healthcare provider could be addressed with a protocol-based postnatal follow-up, to provide time with the healthcare provider to speak about their sexual health. Also, the women thought they should be able to speak to other mothers about their problems, including their sexual health. Four of the participants admitted they felt neglected, as the attention of their clinicians focused exclusively on the baby. Almost all the mothers had doubts about their sexual health; in particular, they feared having pain or uncomfortable feelings during intercourse, they were uncertain about when they should resume sexual activity, and they wanted to know how long it would take for them to return to pre-pregnancy. As such, participants reported seeking information from other sources.

"So, yes, I would have gone to a group meeting but one you could freely not say anything about breastfeeding or babies. Just about all the other doubts we might have or just to explain how we were dealing with the new situation. I think this would be very effective, at least in my case, I would have signed up and I would have appreciated it" (Irene).

## Discussion

Pregnancy is one of the most meaningful life experiences for women [45] and sexual health is an essential part of their quality of life [97]. Similar to the literature about becoming a new mother [31], the women in this study reported the postpartum period was not what they expected. This was especially true in terms of their sexual health. The perceived societal expectations about resuming sexual activity in the postpartum period did not improve their experience, including uncertainty about when to return to sexual activity. From the perspective of the women in this study, sexual activity was defined as intercourse, and anything not considered ‘*normal’* (in the traditional heterosexual context) was stigmatizing. Across the interviews, there were no statements about the relevance of other sexual activities to sexual health apart from intercourse. This is important as Cappell et al. [43] recommends postpartum women consider progressing from non-penetrative sexual activities to intercourse. The women in this study, as also reported by Gómez & Moreno [2], believed their knowledge about the normalcy of sexual health during postpartum was incongruent with societal norms, personal expectations, and lived experiences. When attempting to resume intercourse, most women noted the experience was painful and uncomfortable. This seems to be common as two out of every three women experience dyspareunia when resuming intercourse [46]. The prevailing evidence suggests women can resume intercourse about six to eight weeks after childbirth [44, 46, 58, 98]; however other studies [43, 99] reported sexual activities other than intercourse can be resumed as early as one to two weeks. Although the women in this study believed they needed more time to return to intercourse, they did not try other types of sexual activity, such as masturbation or oral sex.

Several physical factors influence how women experience the resumption of intercourse in the postpartum period. For example, McDonald and Brown [99] reported women with perineal injury can take as long as a year to resume intercourse, although they can engage in other types of sexual activities. The time to resume vaginal intercourse is strongly influenced by physical fatigue, emotional lability, body image, and emotional stressors [100]. The fatigue, accompanied by stress, resulted in the women in this study expressing their need for private space and personal time to relax and recover. Increased time and more space might explain why women are more emotionally satisfied and sexually active when their partners shared household responsibilities [31].

Similar to other studies [44, 59, 97], the women in this study questioned their ability to continue breastfeeding due to fatigue; also, they associated this with a delayed return to intercourse and decreased frequency. Evidence indicates breastfeeding results in sexual impairment associated with dyspareunia for about three to six months [44, 56, 101]. During lactation, vaginal lubrication is reduced due to the high prolactin and low oxytocin levels [102]. This is an important reason for clinicians to engage women in discussions about the basic physiologic changes associated with breastfeeding that impact sexual health.

Disruption of body image was another factor directly influencing the way women perceived their sexual health as well as their satisfaction with intercourse during the postpartum period. Although the women did not feel rejected by their partners, their dissatisfaction with their physical appearance resulted in them feeling less intimate and less confident about returning to sexual activity. Similarly, O’Malley et al. [56] found a positive self-image improves sexual health, including satisfaction with intercourse in the postpartum period. The largely heteronormative society requires mothers to appear young, attractive, and sexually appealing [103–105]. The social construction(s) surrounding sexual attractiveness, gender, traditionally feminine notions of sexuality, and the sexual body influences the body image, self-image, and sexual health of mothers [106, 107].

Sexual health knowledge during pregnancy impacts the sexual attitudes of women in the postpartum period. For example, knowledge deficits about sexuality during pregnancy are associated with women reporting reduced desire and negative attitudes about intercourse [108]. Similarly, knowledge deficits about when and how to return to sexual activity results in women having vaginal intercourse despite not feeling ready because they want to satisfy their partners [22]. In this regard, the women in this study were concerned about their partners feeling neglected as the rationale for having intercourse prior to feeling ready. Although the literature indicates most women experience decreased desire, increased pain, and problems achieving orgasm with sexual intercourse during the first three months postpartum, this progressively resolves by six months for most women [109]. During this time, however, women feel a ‘duty’ to engage in sexual activity despite little desire or reservations [54].

Although the participants were not certain about the right time to return to sexual activity, the women were also not sure who to ask as speaking about sexual activity was perceived to be taboo, also reported in a study by Aribi and colleagues [58]. In this case, poor communication specific to problems with sexual health continues to contribute to decreased quality of life [110]. For example, the inability or unwillingness of women, as well as some men [111], to speak about their sexual health concerns during pregnancy, including the postpartum, results in sexual dysfunction and decreased quality of life [112]. Similar to women in other countries [113], the women in this study constantly worried that their sexual experiences were different from other women or their experiences were ‘not normal’ for mothers. Generally, the women believed their ability to speak openly and honestly about their sexual health would have resulted in a better quality of life.

In retrospect, the women expressed dissatisfaction with the health services they received because their sexual health was neglected. As recommended by Pauleta et al. [45], women need to be able to speak openly with their clinician about their sexual health and to be clearly informed about the changes they should expect. From a feminist post-structuralism perspective, power can be expressed through silence as clinicians ignore the beliefs and preferences of the women [114, 115]. This may explain why the women in this study preferred to seek information from the internet rather than speak to their clinicians. Similar to other reports [60], the women noted their care was always focused on the baby, and not them during the postpartum period. Since consultations are usually more frequent in the antenatal than the postpartum period [98], there might be less time available during the postpartum visits to address sexual health. Overall, the process to return to sexual health was independently managed alone, with the women engaged in experiential learning by ‘trial and error’.

With sexual health evolving during pregnancy [21], this needs to be included as part of the health history [109] as well as evidence-based education provided to the women [113]. In addition, MacAdam, Huuva, & Bertero [116] recommended partner education is very important as the couple need to be involved in discussions about their mutually dependent sexual health in the postpartum period. Yet, addressing concerns about sexual health can be difficult for nurses [117, 118], midwives [53, 113], and physicians [36, 119, 120]. Although sexual health is avoided by clinicians [121, 122], interactive postpartum sexual health education [123] was demonstrated in a randomized control trial of women in the postpartum period (*n* = 250) to be an effective intervention to stimulate conversations and to advance knowledge development.

Finally, in this study, the traditional hetero-normative relationship was observed in the role dynamics of the couple. Although unaware in the postpartum period, the women recognized during the interviews many external societal influences shaped their feminine identity. There is little evidence about this phenomena except for O’Malley et al. [8] noting the positive aspects of a good intimate relationship were influenced by the father adjusting to the baby’s arrival, sharing the household responsibilities, and participating in the childcare. When reflecting about how their partners coped with their new role as mother, the women were thankful and grateful for the patience and understanding provided by their partners. Yet, they also complained about being responsible for everything in the household. These mixed feelings might indicate the women blamed themselves for the problems in the postpartum period rather than accepting these as the responsibility of the couple. Evidence suggests dominant male sexuality resulting in heteronormative cultures results in women suppressing their sexual preferences with decisions in favor of their male partners [124, 125]. Consequently, the situation can lead to an imbalance of power and possibly conflicts manifesting as relationship issues.

As evidence-based practice requires clinicians to incorporate the best evidence from research, consideration should be given to the preferences of the woman in addition to the use of clinical expertise to make care decisions. The human caring practices proposed by Watson [126] can be incorporated into existing guidelines to better manage sexual health throughout pregnancy and postpartum. Instead of avoiding conversations about sexual health [127], clinicians need to offer holistic care including comprehensive sexual assessments [128]. During visits, clinicians need to be authentically present, engaging in active communication, listening to positive and negative experiences, and seeking solutions for identified problems [129]. Finally, postpartum sexual health can be improved by offering optional programs to women that include psychoeducation, skills to enhance sexual satisfaction either within the act of vaginal intercourse or other types of intimate sexually stimulating and satisfying activities, conversations about relationships, and safe spaces for women to discuss their challenges with other women [130].

### Limitations

There are five possible limitations that need to be recognized for this study. First, the sample size was small and focused in a single area of Spain. However, the sample was purposeful to address a gap in the literature specific to postpartum sexual health of Spanish women and the saturation was achieved. Second, this study describes only the reality of primiparous younger well-educated heterosexual women, thereby excluding the experiences of other women. Yet, this limitation is also a strength as the resulting population was homogenous and capable of engaging in selfcare for sexual health. Third, and a common limitation of qualitative evidence, the results should not be generalized outside the Catalan region, or possibly Spain. However, these findings might be applicable for similar sociocultural contexts, and can be aggregated into a metasynthesis [131] in the future with studies from other regions of the world to get a deeper interpretation and expanded knowledge. Fourth, the interview data for analysis was translated from Catalan/Spanish to English. Although a robust process was undertaken to complete this process (described as the eighth step in the methods), the translation still presents an opportunity for errors in coding and categorizing into themes. Fifth and finally, two of the interviews were conducted using Skype® while eight were conducted in person. This has the potential to lose information specific to the non-verbal communication. However, the online route can capture the essence of the interview while permitting more participation from diverse people, enabling participants to feel more comfortable in their environments, increasing the privacy of the conversation, and facilitating the right of participants to immediately leave uncomfortable situations [74, 132, 133].

## Conclusion

Postpartum sexual health is a multifaceted concern for women as they transition from woman to mother, partner to parent, and couple to family. The women in this study were primarily motivated to return to sexual activity to please their partner without guidance from their clinicians. The transition to motherhood needs to include increased awareness about the changes to their bodies, their new responsibilities, and their relationship with partners. Understanding how women experience postpartum sexual health opens a wide range of possibilities to improve a clearly difficult and often confusing experience. In the context of theory guided practice informed by evidence, additional qualitative studies could lead to better understanding of postpartum sexual health in more contexts, cultures, and countries.

## Data Availability

The data used and/or analyzed during the current study are available from the corresponding author on reasonable request.
